# Phenol-like group functionalized graphene quantum dots structurally mimicking natural antioxidants for highly efficient acute kidney injury treatment†

**DOI:** 10.1039/d0sc03246h

**Published:** 2020-08-05

**Authors:** Huan Wang, Dongqin Yu, Jiao Fang, Ya Zhou, Daowei Li, Zhen Liu, Jinsong Ren, Xiaogang Qu

**Affiliations:** State Key Laboratory of Rare Earth Resources Utilization, Laboratory of Chemical Biology, Changchun Institute of Applied Chemistry, Chinese Academy of Sciences Changchun 130022 P. R. China jren@ciac.ac.cn xqu@ciac.ac.cn; University of Science and Technology of China Hefei 230029 P. R. China; Department of Oral Implantology, School and Hospital of Stomatology, Jilin University Changchun 130021 P. R. China; Jilin Provincial Key Laboratory of Tooth Development and Bone Remodeling, School of Stomatology, Jilin University Changchun 130021 P. R. China; Beijing Advanced Innovation Center for Soft Matter Science and Engineering, Beijing University of Chemical Technology Beijing 100029 P. R. China

## Abstract

Acute kidney injury (AKI) is a syndrome characterized by rapid loss of renal excretory function with high in-hospital mortality. The excess generation of reactive oxygen species (ROS) in the kidneys during AKI has been considered a major cause of renal failure. Currently available antioxidants for AKI treatment often lack the required antioxidative efficacy or renal accumulation rate. Herein, inspired by the structure of natural phenolic antioxidants, phenol-like group functionalized graphene quantum dots (h-GQDs) with both high ROS scavenging efficacy and renal specificity are constructed for AKI antioxidative therapy. Similar to natural polyphenols, the abundant phenol-like groups on h-GQDs are demonstrated to be the active components exerting antioxidative effects. Further exhaustive mechanistic investigations indicate that the ultrahigh antioxidative activity of h-GQDs originates not solely from the phenol-like groups, but also from the synergy between adjacent phenol-like groups, as well as the removal of unfavorable carbonyl groups on h-GQDs. In AKI mice, h-GQDs can effectively protect the kidneys from oxidative injury with only a one-sixteenth dose of the clinical antioxidant *N*-acetylcysteine (NAC) and show no evidence of toxicity. The findings of this study will facilitate development of high-performance carbon-based antioxidative platforms *via* structure–activity relationships for treating AKI and other ROS-related diseases.

## Abstract 2

Phenol-like group functionalized graphene quantum dots that structurally mimic natural antioxidants are constructed as high-efficacy reactive oxygen species scavengers for acute kidney injury antioxidative therapy.

## Graphical abstract

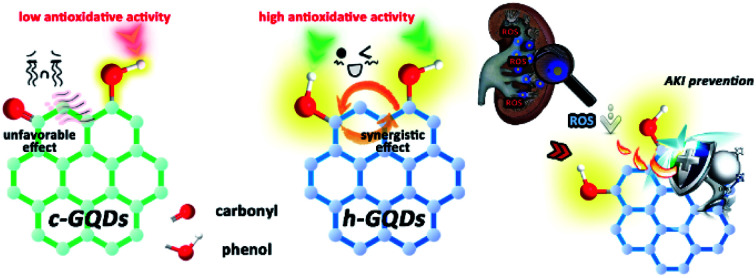

## Introduction

Acute kidney injury (AKI) is a syndrome characterized by the rapid loss of excretory function of the kidneys and is associated with increasing risk of in-hospital death.^1,2^ Although significant achievements have been made to understand the pathogenesis of AKI, no specific therapies have emerged for effective AKI amelioration or expedited recovery. In current clinical settings, only renal replacement therapies (RRTs) are available for AKI amelioration; however, comprehensive kidney support is difficult to achieve.^3,4^ As a result, patients with AKI who are treated with RRT still have a high mortality rate of 50–60%, and nearly 20% of the surviving patients remain on dialysis after hospital discharge. During AKI, the reactive oxygen species (ROS) overproduction has been counted as a major cause of renal failure.^5,6^ Towards this goal, antioxidative therapy *via* efficient ROS scavenging is regarded as one of the critical approaches to protect renal function in AKI. Although small molecular antioxidants have shown positive effects in alleviating contrast agent-induced renal damage, such as *N*-acetylcysteine (NAC), their poor bio-availability and specificity hinder the clinical availability to other types of AKI.^5,7^

Alternatively, ultrasmall nanomaterials with both renal specificity and antioxidative activity have emerged as potential candidates for alleviating AKI.^6–8^ Compared with small molecular drugs, these ultrasmall nanoagents with reasonable sizes can remain in the kidneys for a significantly longer period and have better targeting ability.^9,10^ For instance, antioxidative nanoclusters based on the variable valence state of metal centers have been developed for AKI treatment.^6^ However, these metal-containing nanoagents usually pose some safety concerns associated with their unwanted metal ion leakage and possible residues.^11^ Additionally, metal-free nanoagents such as DNA origami nanostructures and black phosphorus nanosheets have been used to alleviate AKI.^7,12^ However, translation of these nanoagents faces several potential roadblocks including ambiguous *in vivo* stability, indefinite immune responses, and potential acute toxicity.^13,14^ As of now, the engineering of antioxidative nanoagents for treating renal diseases is still in its infancy.^15,16^ Hence, it remains challenging to design novel nanoplatforms for AKI treatment with great antioxidative activity, high renal accumulation, and high biocompatibility.

Owing to their high stability, low toxicity, and facile production on a large scale, nanocarbons have arisen as favorable candidates for biomedical applications.^17^ Recently, a series of carbogenic nanodots, such as graphene quantum dots (GQDs), nitrogen-doped carbon dots, selenium-doped carbon dots, chlorine-doped GQDs, and nitrogen/sulphur-co-doped carbon dots, have emerged as antioxidants for ROS elimination.^18–25^ In particular, GQDs have shown efficient renal clearance properties and long body retention, providing them great kidney targeting ability.^26,27^ Significantly, the biological effects of GQDs functionalized with enriched oxygenated groups have been investigated exhaustively, and they exhibit high biocompatibility both *in vitro* and *in vivo* even at a high dose.^28,29^ However, such GQDs usually have relatively low antioxidative activity, which greatly limits their wide biological usage under various physiological and pathological conditions, let alone developing the antioxidative effect of GQDs for treating AKI. More importantly, the design of high-efficacy GQD-based antioxidative platforms *via* engineering surface oxygenated groups has not been well explored.^21^

**Fig. 1:**
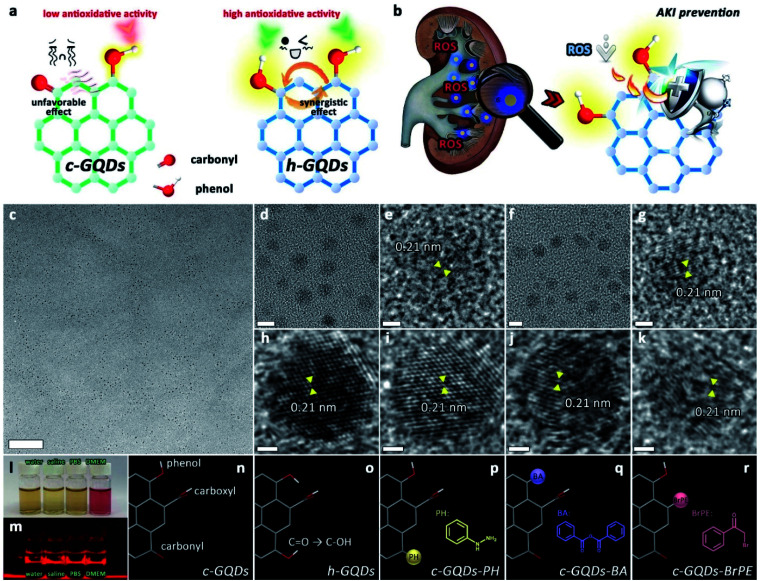
Design and characterization of h-GQDs for AKI treatment. Schematic illustration of antioxidative activity of h-GQDs (a) and their usage as ROS scavengers for treating AKI (b). TEM images of h-GQDs (c–e), p-GQDs (f, g), c-GQDs (h), c-GQDs–PH (i), c-GQDs–BA (j), and c-GQDs–BrPE (k). Scale bars are equal to 100 nm (c), 5 nm (d, f), and 1 nm (e, g–k). Photograph of h-GQDs dissolved in different solutions including water, saline, PBS, and DMEM containing 10% FBS (l). Photograph of the Tyndall effect of h-GQDs dissolved in different solutions (m). Schematic illustrations of the chemical structures of c-GQDs (n), h-GQDs (o), c-GQDs–PH (p), c-GQDs–BA (q), and c-GQDs–BrPE (r). Red bands indicate O atoms, white bands indicate H atoms, and gray bands indicate C atoms. Insets: the molecular structure of PH (p), BA (q), and BrPE (r).

As important natural antioxidants, polyphenols show pronounced biological effects including high physiological and pharmacological activities. Generally, the phenolic groups of polyphenolic antioxidants act as H-atom donors for ROS trapping.^30,31^ A phenolic H-atom is captured by a free radical, leading to the formation of an intermediate phenoxyl radical. The phenoxyl radical so formed is resonance-stabilized by p–π orbital overlap until it encounters a further free radical with which it couples rapidly.^30^ Additionally, the antioxidative activity of polyphenols could be greatly influenced by various substituent groups. Generally, there are various oxygenated groups, such as hydroxyl (C–OH), carbonyl (C=O), and carboxyl (COOH), on GQDs. The C–OH species can attach either to the sp^2^ carbon centers or the sp^3^ carbon centers, and the sp^2^ carbon center-bonded C–OH species on the graphene structure are termed “phenol-like groups”.^32–35^ Inspired by these principles, we envision that the phenol-like groups on the graphene structure can act as H-atom donors for ROS trapping, and the related intermediate radicals could be well stabilized by the long-range π-electron system. Meanwhile, other surface oxygenated groups may have positive or negative effects on the antioxidative activity. Herein, we present phenol-like group functionalized graphene quantum dots (h-GQDs) with preferential renal accumulation as high-efficacy ROS scavengers for treating AKI (Fig. 1b). As expected, the phenol-like functionalities on h-GQDs were demonstrated to be the active components for the antioxidative activity. Further exhaustive mechanistic investigations indicate that the ROS scavenging capacity of the phenol-like groups on the graphene structure can be greatly suppressed by nearby carbonyl groups and significantly enhanced by other adjacent phenol-like moieties (Fig. 1a). Therefore, the observed high antioxidative activity of h-GQDs originates from the well-tailored specific oxygenated groups including increased amounts of phenol-like groups and decreased amounts of carbonyl groups. Benefitting from their reasonable sizes, these h-GQDs show high renal accumulation in AKI mice over a long period of time (>72 h). As a result, these h-GQDs can exert comparable therapeutic effects with only a one-sixteenth dose of the clinical antioxidant *N*-acetylcysteine (NAC) and show no evidence of toxicity. We expect that this study could facilitate the development of high-efficacy carbon-based antioxidative platforms *via* structure–activity relationships to address the practical needs of treating AKI and other ROS-related diseases.

## Results and discussion

**Fig. 2:**
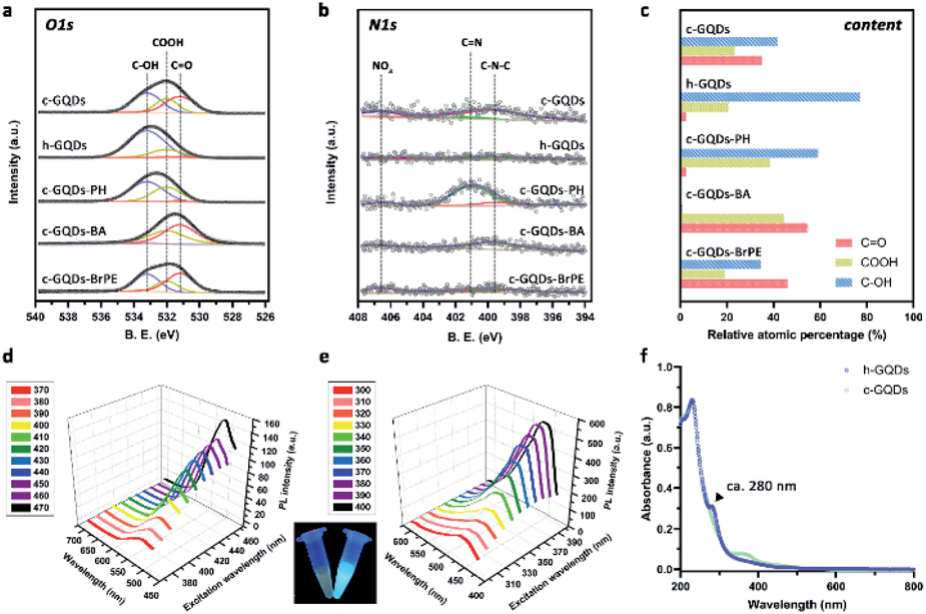
Characterization of the distribution of different oxygenated groups on various GQDs. O1s (a) and N1s (b) XPS analysis of various GQDs. The atomic percentage of different oxygenated groups on various GQDs are calculated *via* the deconvolution of the XPS spectra (c). PL spectra of c-GQDs (d) and h-GQDs (e) under different excitations. Inset: photograph of the c-GQD (left) and h-GQD (right) aqueous solutions obtained under UVA light. UV-Vis-NIR spectra of c-GQDs and h-GQDs (f).

Classical GQDs (c-GQDs) with enriched oxygenated groups including C=O, C–OH, and COOH were first prepared as precursors.^36^ As an efficient reductant, NaBH_4_ could selectively reduce C=O to C–OH and significantly increase the amount of C–OH on carbon without reducing other species.^37–39^ In our present design, C=O on c-GQDs was efficiently converted to C–OH to result in phenol-like group functionalized graphene quantum dots (h-GQDs) by using an appropriate amount of NaBH_4_ as the reductant. For exploration of the detailed roles of various oxygenated groups in the antioxidative activity, phenylhydrazine (PH), benzoic anhydride (BA), and 2-bromo-1-phenylethanone (BrPE) were employed as high-specificity deactivating agents to react with C=O, C–OH, and COOH on c-GQDs, which were defined as c-GQDs–PH, c-GQDs–BA, and c-GQDs–BrPE, respectively (Fig. S1†).^36,40^ As shown in Fig. 1c–k, transmission electron microscope (TEM) images demonstrated that there were negligible differences in size and morphology among various GQDs. All these GQDs were extremely homogeneous with an average size of 4.4–4.8 nm (Fig. S2†). High-resolution TEM (HR-TEM) images indicated that all the GQDs had fine crystallinity with a lattice spacing of 0.21 nm, which could be attributed to the characteristic (102) diffraction planes of the sp^2^ graphitic carbon (Fig. 1e, g, h–k). High-resolution O1s X-ray photoelectron spectroscopy (XPS) was used to explore the distribution of different oxygenated groups on various GQDs in detail. As shown in Fig. S3a,† the total oxygen content of h-GQDs decreased only slightly after reduction. As shown in Fig. 2a, pristine c-GQDs were mainly covered with C=O, C–OH, and COOH species. After reduction, both the dramatic decrease in intensity of the C=O signal and the significant increase in intensity of the C–OH signal could be observed, and only some COOH was removed. Notably, the content of C–OH accounted for nearly three-quarters of all oxygenated groups on h-GQDs (Fig. 2c). These results demonstrated that the C=O was efficiently converted to C–OH during the reduction process. Fourier transform infrared spectroscopy (FT-IR) was further carried out to confirm the formation of new functional groups on h-GQDs. Compared with c-GQDs, h-GQDs showed a significant decrease of the C=O signal at 1710 cm^−1^ and an obvious increase of the C–OH signal at 3415 cm^−1^ (Fig. S3b†). Importantly, a new vibrational band at 1200 cm^−1^ could be detected and could be assigned to the typical C–O stretching of phenolic moieties on the graphene structure.^33^ Such results indicated that the abundant newly formed C–OH species on h-GQDs contained phenol-like moieties. Then, the optical properties of c-GQDs and h-GQDs were investigated to reveal their surface chemistry. In the UV-Vis-NIR absorption spectra, the absorption peak at *ca.* 370 nm of c-GQDs disappeared after NaBH_4_ reduction, and a new absorption peak at *ca.* 280 nm appeared (Fig. 2f).^38^ In the photoluminescence (PL) spectra, the c-GQDs showed PL excitation (PLE) and PL peaks at 470 and 515 nm, respectively (Fig. 2d and S4a†). As for h-GQDs, blue-shifted PLE and PL peaks at 400 and 465 nm were observed (Fig. 2e and S4b†). It has been known that the phenol-like functionalities on GQDs contributed the most to the blue emissions and the luminescence enhancement, whereas the carbonyl groups on GQDs contributed to the green emissions.^34,35,38,41,42^ These results collectively demonstrated the removal of carbonyl groups and the functionalization of phenol-like groups on c-GQDs during the reduction process. Then, XPS analysis was used to verify the occurrence of the deactivation process of various oxygenated groups on c-GQDs. Compared with c-GQDs, the decrease in the intensities of the C–OH signal for c-GQDs–BA and C=O signal for c-GQDs–PH and the increase in the C=O signal for c-GQDs–BrPE could be clearly detected in the O1s XPS spectra, indicating that the three reactions had indeed occurred (Fig. 2a).^36^ Additionally, the presence of the N1s peak in c-GQDs–PH could be ascribed to the formation of C=N bonds (Fig. 2b). We further identified the *ζ* potentials of various GQDs. As shown in Fig. S5,† the *ζ* potential value of h-GQDs was higher than that of c-GQDs. Considering that there was no obvious change in the total oxygen content of h-GQDs after reduction, the increased *ζ* potential value of h-GQDs could be attributed to the removal of negatively charged C=O. Moreover, the removal of COOH from c-GQDs by BrPE could lead to the formation c-GQDs–BrPE with a higher *ζ* potential value. The chemical structures of various GQDs are illustrated in Fig. 1n–r. All these results demonstrated the successful construction of phenol-like group functionalized h-GQDs and the occurrence of the deactivation process of various oxygenated groups on c-GQDs. Benefitting from the enriched oxygenated groups, all these GQDs showed excellent solubility in water and physiological solutions, and distinct Tyndall effects could be well observed (Fig. 1l and m, and S6†).

**Fig. 3:**
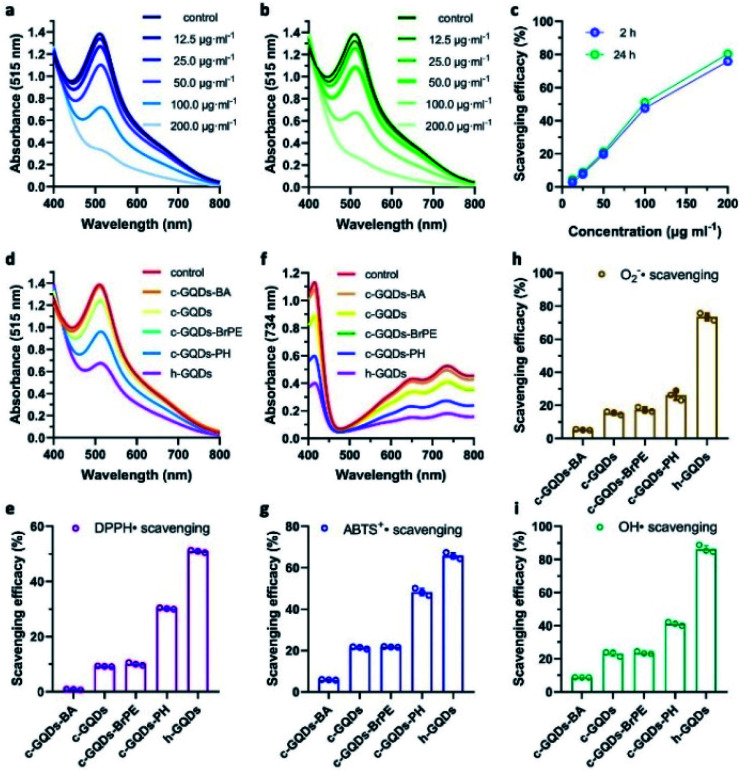
Antioxidative activity of h-GQDs and related mechanisms. UV-Vis-NIR spectra of DPPH˙ co-incubated with different concentrations of h-GQDs for 2 h (a) and 24 h (b). Concentration-dependent scavenging efficacies for DPPH˙ of h-GQDs after 2 h and 24 h of co-incubation (c). UV-Vis-NIR spectra of DPPH˙ co-incubated with various GQDs for 24 h (d). Scavenging efficacies for DPPH˙ of various GQDs after 24 h of co-incubation (e). The concentrations of various GQDs are 100 μg mL^−1^. UV-Vis-NIR spectra of ABTS^+^˙ co-incubated with various GQDs for 0.5 h (f). Scavenging efficacies for ABTS^+^˙ of various GQDs after 0.5 h of co-incubation (g). The concentrations of various GQDs are 20 μg mL^−1^. Scavenging efficacies for superoxide radicals (h) and hydroxyl radicals (i) of various GQDs. The concentrations of various GQDs are 5 μg mL^−1^. Error bars represent the standard deviation from the mean (*n* = 3).

The DPPH˙ assay was used to investigate the H-atom donating ability of h-GQDs for antioxidative activity. The change of absorbance of DPPH˙ at 515 nm was monitored after co-incubating with h-GQDs for 2 h. As shown in Fig. 3a, these h-GQDs exhibited extremely high antioxidative activity in a concentration-dependent manner. As shown in Fig. 3b and c, the DPPH˙ scavenging efficacies did not show obvious changes when the co-incubation time was extended to 24 h, suggesting that most of the labile H-atoms on h-GQDs were exhausted within 2 h of reaction time. Compared with c-GQDs, these h-GQDs exhibited at least 5 times higher activity under the same experimental conditions (Fig. 3d and e). We further explored the DPPH˙ scavenging efficacies of various GQDs with deactivated oxygenated groups to understand the contribution of different oxygenated groups toward the activity. Compared with c-GQDs, the activity of c-GQDs–BA dropped by about 90%, indicating that the antioxidative activity of c-GQDs comes mainly from C–OH species (Fig. 3d and e). Moreover, the activity of c-GQDs–BrPE was similar to that of c-GQDs. This is because the carboxyl groups and nonphenolic hydroxyl groups are usually not able to serve as H-atom donors like phenolic groups for ROS trapping, which also suggests that the phenol-like groups on h-GQDs are the active components exerting antioxidative effects. Significantly, c-GQDs–PH exhibited obviously higher activity than c-GQDs, demonstrating that the presence of C=O could have unfavorable effects on the activity. Such results indicated that the observed high antioxidative activity of h-GQDs should not originate solely from the phenol-like moieties.

**Fig. 4:**
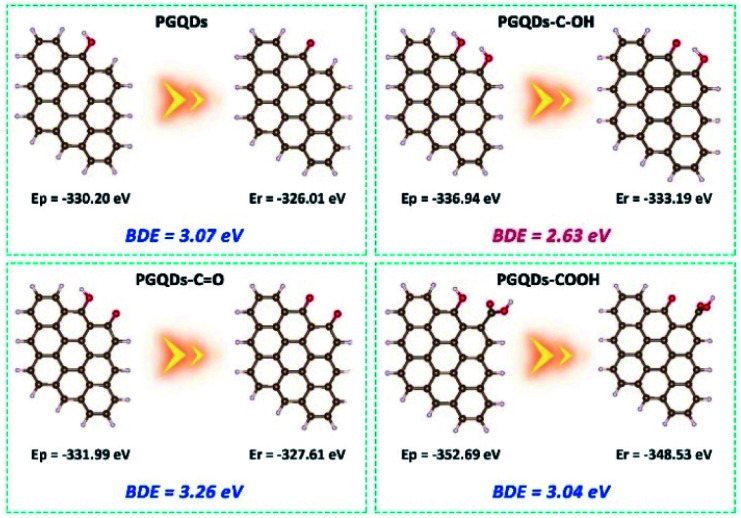
DFT calculations. The DFT results of the influence of various adjacent oxygenated groups on the O–H bond dissociation energy (BDE) of the phenol-like group on the graphene structure. A relatively low O–H BDE facilitates the H-abstraction reaction between the antioxidant and the radical. Comparison of the O–H BDEs of the phenol-like group on the graphene structure before (PGQDs) and after adding an adjacent phenol-like group (PGQDs–C–OH), a carbonyl group (PGQDs–C=O), or a carboxyl group (PGQDs–COOH) on the carbon surface. Red bands indicate O atoms, white bands indicate H atoms, and brown bands indicate C atoms. BDE = *E*_r_ + *E*_h_ − *E*_p_, in which *E*_r_ is the energy for radicals generated after H abstraction from the parent graphene structure, *E*_h_ is the energy for the hydrogen atom, −1.12 eV, and *E*_p_ is the energy for the parent graphene structure.

For natural phenolic antioxidants, the antioxidative activity could be greatly influenced by various ring substituents.^30^ To explore the real origin of the observed high activity of h-GQDs, the O–H bond dissociation energy (BDE) of the phenol-like group on the graphene structure (denoted as PGQDs) was studied by DFT calculations (Fig. 4). Generally, a relatively low O–H BDE of the phenolic moiety facilitates H-atom donation, suggesting a high antioxidative activity. The possible effects of various oxygenated groups on the activity of the phenol-like group on PGQDs were investigated by adding one specific oxygenated group (carbonyl, carboxyl, or phenol-like group) around the phenol-like group, which were denoted as PGQDs–C=O, PGQDs–COOH, and PGQDs–C–OH, respectively. The BDE of the phenol-like group on PGQDs and PGQDs–COOH could be calculated to be 3.07 eV and 3.04 eV, whereas the BDE for PGQDs–C=O was 3.26 eV. According to these results, the H donating ability of the phenol-like group on PGQDs could be greatly suppressed by the nearby carbonyl group. In addition, the approaching of the carboxyl group to the phenol-like group does not much affect the H-atom donating ability, consistent with our experimental results. Significantly, PGQDs–C–OH had the lowest BDE value of 2.63 eV, indicating that the energy barrier for O–H bond dissociation of the phenol-like group on PGQDs could be effectively reduced by other adjacent phenol-like groups.

Comprehensive insight into the antioxidative activity of h-GQDs and the related mechanisms could be summarized as follows. As the active sites, the phenol-like groups could not only serve as H-atom donors to exert antioxidative effects, but also synergistically enhance the H-atom donating ability of other adjacent phenol-like groups and thus the antioxidative activity. However, the existence of carbonyl groups could decrease the H-atom donating ability of nearby phenol-like groups, causing unfavorable effects on the activity. Hence, h-GQDs with abundant phenol-like groups and negligible carbonyl groups exhibited extremely high antioxidative activity. Furthermore, we performed assays with ABTS^+^˙, superoxide radicals (˙O_2_^−^), and hydroxyl radicals (˙OH) to re-confirm the antioxidative properties of h-GQDs. As shown in Fig. 3f–i, h-GQDs could effectively eliminate ABTS^+^˙, ˙O_2_^−^ and ˙OH under physiological conditions.^43^

**Fig. 5:**
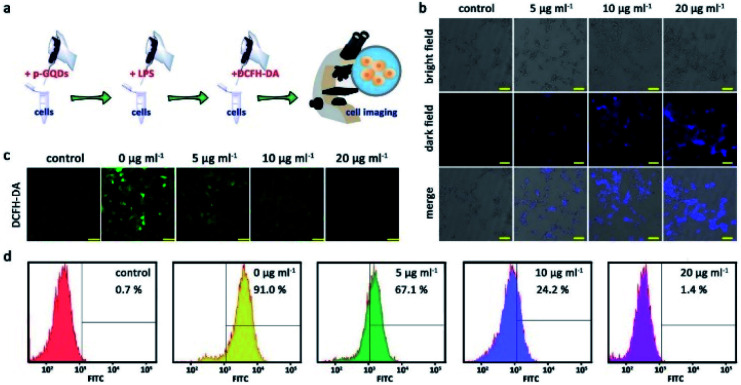
*In vitro* ROS scavenging activity of p-GQDs. Schematic illustration of the introduction of excess intracellular ROS stimulated by LPS and the related *in vitro* ROS scavenging process by using p-GQDs (a). Concentration-dependent cellular internalization imaging of p-GQDs (b). Scale bars are equal to 50 μm. Concentration-dependent fluorescence imaging (c) and flow cytometry analysis results (d) of ROS scavenging efficacy of p-GQDs in HEK-293T cells by using DCFH-DA as the typical fluorescent sensor. From left to right: cells treated with DCFH-DA alone; cells treated with LPS and DCFH-DA; cells treated with LPS, DCFH-DA, and p-GQDs (5 μg mL^−1^); cells treated with LPS, DCFH-DA, and p-GQDs (10 μg mL^−1^); cells treated with LPS, DCFH-DA, and p-GQDs (20 μg mL^−1^). Scale bars are equal to 50 μm.

Considering their bio-related usages, h-GQDs were then non-covalently functionalized with PEG molecules (molecular weight: 1000) *via* the hydrophobic interaction between h-GQDs and terminal phospholipid groups of mPEG–DSPE, which were termed p-GQDs.^44^ Ultrasmall nanomaterials functionalized with PEG with a suitable molecular weight can retain their efficient renal clearance properties and provide a relatively longer body retention.^45,46^*ζ* potential measurements of p-GQDs indicated successful PEGylation, which was re-confirmed by FT-IR analysis (Fig. S3d and S5†). Moreover, h-GQDs and p-GQDs showed similar UV-Vis-NIR absorption spectra, indicating that the h-GQD cores were well preserved by PEGylation (Fig. S3c†). As shown in Fig. 1f and g, and S2c,† TEM images and size distribution analysis indicated that there were negligible differences in size and morphology between h-GQDs and p-GQDs. Significantly, the non-covalent PEGylation showed negligible influence on the antioxidative activity of h-GQDs (Fig. S7†). We then explored the antioxidative activity of p-GQDs *in vitro* by using HEK-293T cells, a typical human embryonic kidney cell line (Fig. 5a). The cytotoxicity of p-GQDs was evaluated at first. As shown in Fig. S8,† the MTT assay revealed that all the cell viabilities were not hindered by p-GQDs even at the highest concentration of 200 μg mL^−1^ after 24 h of co-incubation. Cellular uptake of p-GQDs could be visualized by fluorescence imaging. As expected, p-GQDs could be efficiently taken up by HEK-293T cells after 2 h of co-incubation in a concentration-dependent manner (Fig. 5b). Afterwards, LPS was used to stimulate cells to produce excess intracellular ROS, which could be evaluated by using DCFH-DA as a typical fluorescence indicator. As shown in Fig. 5c, cells treated with LPS revealed increased intracellular ROS levels. Upon the treatment of p-GQDs, it was clearly found that p-GQDs could effectively alleviate oxidative stress *in vitro* in a concentration-dependent manner, which was also re-confirmed by flow cytometry analysis (Fig. 5d).

**Fig. 6:**
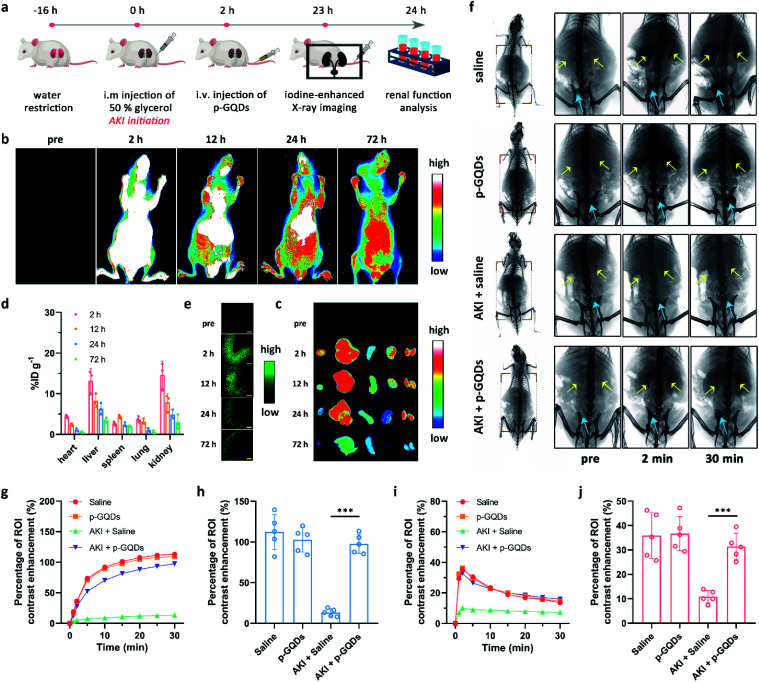
Bio-distribution and therapeutic effect of p-GQDs in AKI mice. Schematic illustration of the preparation of a murine model with rhabdomyolysis-induced AKI and related *in vivo* antioxidative therapy of AKI by using p-GQDs (a).Time-dependent *in vivo* (b) and *ex vivo* (c) fluorescence imaging of AKI mice after i.v. injection of Cy5.5-p-GQDs. From left to right in (c): the heart, liver, spleen, lungs, and kidneys. Bio-distribution of Cy5.5-p-GQDs from AKI mice after i.v. injection (d).Time-dependent fluorescence imaging of kidney cryosections from AKI mice after i.v. injection of Cy5.5-p-GQDs (e). Relative output is pseudo-colored in green for clear visualization. Scale bars are equal to 500 μm. Error bars represent standard deviation from the mean (*n* = 3). Representative X-ray images of healthy mice treated with saline, healthy mice treated with p-GQDs, AKI mice treated with saline, and AKI mice treated with p-GQDs at 2 and 30 min after i.v. injection of iopromide (f). Yellow arrows indicate the kidneys and blue arrows indicate the bladder. Time-dependent ROI analysis of X-ray intensity variation (percentage of contrast enhancement) in the bladder (g) and kidneys (i) of mice after various treatments. ROI analysis of X-ray intensity variation in the mouse bladder at 30 min after i.v. injection of iopromide under different treatments (h). ROI analysis of maximal X-ray density variation in the kidneys of mice after various treatments (j). Error bars represent standard deviation from the mean (*n* = 5). Asterisks indicate statistically significant differences (*P < 0.05, **P < 0.01, and ***P < 0.001).

We then explored the bio-distribution of p-GQDs in AKI mice. A murine model of rhabdomyolysis-induced AKI was established *via* intramuscular (i.m.) injection of glycerol into dehydrated healthy mice at first (Fig. 6a).^7^ This time point was defined as the initiation of AKI. Time-dependent *in vivo* and *ex vivo* fluorescence imaging was carried out to monitor the bio-distribution of p-GQDs visually. The excitation wavelength of h-GQDs was around 400 nm with low penetration capacity, which was not suitable for bio-imaging studies. Thus, h-GQDs were non-covalently functionalized with DSPE–PEG-Cy5.5 to result in fluorescent p-GQDs (Cy5.5-p-GQDs).^44^ The successful construction of Cy5.5-p-GQDs was confirmed *via* PL spectra (Fig. S9†). 2 h after initiation of the AKI model, Cy5.5-p-GQDs were intravenously injected into the mice. Cy5.5-p-GQDs could be easily found in injured kidneys after intravenous (i.v.) injection in AKI mice (Fig. 6b and c). Significantly, fluorescence signals from Cy5.5-p-GQDs could also be detected in the kidneys even at 72 h post-injection, suggesting their long renal retention. We further explored the bio-distribution of Cy5.5-p-GQDs *via* the quantitative analysis of fluorescence signals achieved from the tissue homogenates *ex vivo*.^47,48^ As shown in Fig. 6d, most of these Cy5.5-p-GQDs still remained in the kidneys at 72 h post-injection, which could be ascribed to the efficient renal accumulation of these p-GQDs. Fig. 6e additionally describes the time-dependent accumulation of Cy5.5-p-GQDs in the kidneys by using confocal images of cryosections harvested from AKI mice after i.v. injection. All these results strongly evidenced the efficient renal accumulation properties and relatively long renal retention of p-GQDs in AKI mice and their great potential for further *in vivo* AKI treatment.

We then sought to use p-GQDs to alleviate AKI *in vivo*. Mice were randomly divided into four groups, which were defined as healthy mice + saline, healthy mice + p-GQDs (10 mg kg^−1^), AKI mice + saline, and AKI mice + p-GQDs (10 mg kg^−1^). Afterwards, mouse renal function was evaluated by iopromide-enhanced X-ray imaging at one day post-initiation of the AKI model.^10,49^Fig. 6f demonstrates that contrast enhancement of the kidneys in the groups of healthy mice + saline and healthy mice + p-GQDs reached the maximum peak within 2 min post-injection of iopromide and decreased dramatically over time, and the gradual bladder accumulation of iopromide was also clearly observed. The above results not only indicated that p-GQDs exhibited excellent safety, but also provided standard data for the following assessment of antioxidative therapy. In the group of AKI mice + saline, significantly decreased accumulation of iopromide was found in the kidneys with little excretion of iopromide to the bladder. By contrast, AKI mice treated with p-GQDs exhibited obviously recovered renal functions, and all the dynamic curves of iopromide in the kidneys and bladder were similar to those of healthy mice treated with saline or p-GQDs (Fig. 6g–j). These results indicated that p-GQDs could serve as efficient nanoagents to recover renal function in AKI mice.

**Fig. 7:**
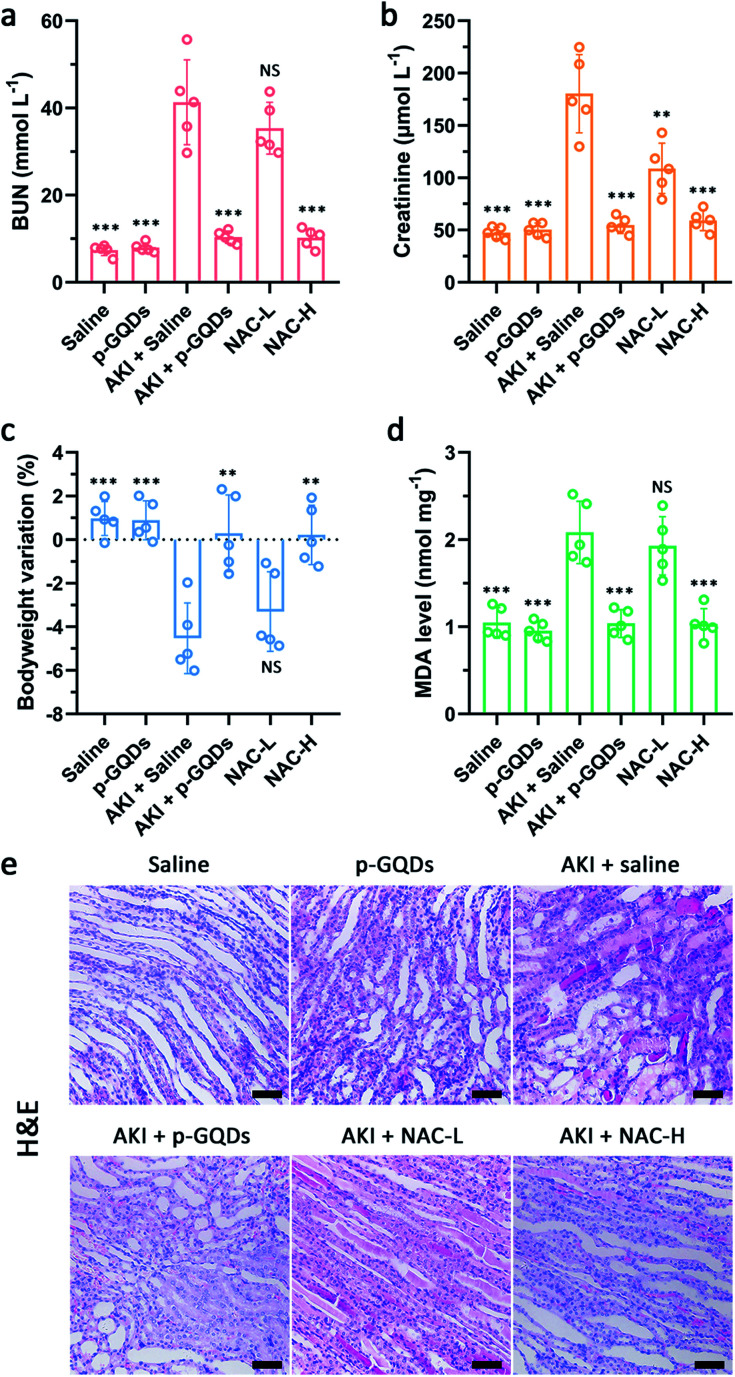
Blood serum analysis and histopathological analysis of renal tissue after AKI treatment. BUN levels (a) and creatinine levels (b) in the blood after various treatments one day post-initiation of AKI. Bodyweight changes of mice after various treatments one day post-initiation of AKI (c). MDA levels in the homogenates of the kidneys from mice after various treatments one day post-initiation of AKI (d). H&E staining images of kidney tissues collected from mice after various treatments one day post-initiation of AKI (e). Scale bars are equal to 50 μm. Error bars represent standard deviation from the mean (*n* = 5). Asterisks indicate statistically significant differences (*P < 0.05, **P < 0.01, and ***P < 0.001).

To verify the practical therapeutic efficacy of p-GQDs for treating AKI, NAC and PEGylated c-GQDs were selected and used as a positive control. In our experimental design, therapeutic efficacies of p-GQDs (10 mg kg^−1^), PEGylated c-GQDs (10 mg kg^−1^), high-dose NAC (NAC-H, 160 mg kg^−1^), and low-dose NAC (NAC-L, 10 mg kg^−1^) were investigated in detail. Generally, casts formed by denatured protein precipitation in renal tubules were utilized as characteristic indicators for kidney disease. As shown in Fig. 7e, S10e, and S11,† treatment effects based on hematoxylin and eosin (H&E) staining images indicated that damaged renal tubules and casts could be clearly observed in the kidney sections in the saline, NAC-L, and PEGylated c-GQD treatment groups while much fewer damaged structures were found in the p-GQD and NAC-H treatment groups, and nearly no damage was found within 7 days after p-GQD treatment. High levels of serum creatinine and blood urea nitrogen (BUN) were the clinical manifestations of the accumulation of end products of nitrogen metabolism, which could aid the evaluation of renal function in clinics. The levels of creatinine and BUN of AKI mice re-confirmed that p-GQDs and NAC-H could efficiently recover the renal function of AKI mice, while NAC-L and PEGylated c-GQDs did not produce obvious therapeutic effects (Fig. 7a and b, S10a and b, and S12a and b†). Moreover, the survival curve and body weight of AKI mice were also monitored, further suggesting that p-GQDs were highly efficient for AKI treatment (Fig. 7c, S10c, S13, and S14†). Generally, excess ROS generation could induce lipid peroxidation. Therefore, we further explored whether p-GQDs could relieve lipid peroxidation in the injured kidneys by using the malondialdehyde (MDA) assay. Indeed, lipid peroxidation of the injured kidneys in AKI mice could be relieved well by p-GQDs (Fig. 7d, S10d, and S12c†). Taken together, these results demonstrated the feasibility of p-GQDs as efficient antioxidants to protect kidneys from injury during AKI. Notably, benefitting from their high antioxidative activity, p-GQDs provided a much better therapeutic efficacy compared with PEGylated c-GQDs. Significantly, p-GQDs with a relatively low dose of 10 mg kg^−1^ had similar therapeutic effects to high-dose NAC (160 mg kg^−1^), further indicating the admirable therapeutic effect of p-GQDs even at a low dose.

Given their excellent efficacy for AKI treatment, we then explored the biocompatibility and systemic toxicity of p-GQDs in healthy mice. Compared with the control group, mice in the test group showed negligible differences in body weight, eating, drinking, and activity during the whole experimental period (Fig. S15†). H&E staining images of major organs including the heart, liver, spleen, lungs, and kidneys from mice 15 days after different treatments demonstrated that p-GQDs had high biocompatibility and negligible systemic toxicity (Fig. S16†). The results of hematology analysis and blood biochemical assay revealed that there were no significant differences between the test group and the control group, and all the parameters fell well within the reference index (Fig. S17–S20†).

## Conclusions

In summary, phenol-like group functionalized graphene quantum dots (h-GQDs) were developed as high-efficacy ROS scavengers for treating AKI. These h-GQDs exhibited ultrahigh antioxidative activity and could scavenge multiple cytotoxic ROS. Mechanistically, the synergy between the functionalized phenol-like moieties as well as the removal of unfavorable carbonyl groups on h-GQDs collectively contributed to the observed high activity. The results of *in vivo* experiments demonstrated that the superb antioxidative activity, high renal accumulation, and excellent biocompatibility afforded these h-GQDs great potential for AKI treatment towards practical usage. We envision that this study could offer valuable insight into the design of high-performance carbon-based antioxidative platforms *via* structure–activity relationships for treating AKI and other ROS-related diseases.

## Ethical statement

All animal experiments were performed in accordance with the NIH guidelines for the care and use of laboratory animals (NIH Publication No. 85-23 Rev. 1985) and approved by the Jilin University Animal Care and Use Committee. Balb/c mice (8–10 weeks, 25 g) were obtained from the Laboratory Animal Center of Jilin University (Changchun, China), and all animal care and handling procedures were in accordance with the guidelines approved by the ethics committee of Jilin University.

## Conflicts of interest

The authors declare no competing interests.

## Supplementary Material

SC-011-D0SC03246H-s001
