# Supplementary material for: Phenol-like group functionalized graphene quantum dots structurally mimicking natural antioxidants for highly efficient acute kidney injury treatment
Source: Chem Sci. 2020 Aug 5;11(47):12721–30. doi: 10.1039/d0sc03246h (PMC8163143; doi:10.1039/d0sc03246h)
Supplement: SC-011-D0SC03246H-s001 [file SC-011-D0SC03246H-s001.pdf]

Electronic Supplementary Material (ESI) for Chemical Science.
This journal is © The Royal Society of Chemistry 2020

Electronic Supplementary Information (ESI) for *Chemical Science*

Phenol-Like Groups Functionalized Graphene Quantum Dots Structurally Mimicking Natural Antioxidants for Highly Efficient Acute Kidney Injury Treatment

Huan Wang, Dongqin Yu, Jiao Fang, Ya Zhou, Daowei Li, Zhen Liu, Jinsong Ren and Xiaogang Qu

Ⅰ. Methods

Chemicals. HCl, HNO_3, H_2SO_4, $K_2S_2O_8$, $CHCl_3$, and Na_2CO_3 were purchased from Beijing Chemicals, Inc.. The mPEG-DSPE (M_W = 1000) and DSPE-PEG-Cy5.5 (M_W = 1000) were ordered from ToYong Biotechnology. $NaBH_4$, 2,2'-azinobis (3-ethylbenzothiazoline 6-sulfonate) (ABTS), benzoic anhydride (BA), phenylhydrazine (PH), and 2-bromo-1-phenylethanone (BrPE) were purchased from Sigma Aldrich. 1,1-diphenyl-2-picrylhydrazyl free radical (DPPH·) were purchased from Alfa Aesar. Ultrapure (UP) water was prepared by Milli-Q-Plus system (18.2 MΩ cm^{-1}) and used through all the experiments. Dialysis bags (molecular weight cut off: 1 kDa and 3.5 kDa) were ordered from Spectrumlabs. All other reagents were of analytical grade and used as received.

Synthesis of c-GQDs. c-GQDs were prepared according to previous reports.[1] Briefly, GO was synthesized from graphite by a modified Hummers method.[2] Afterwards, the solution of GO (120 mL, 0.5 mg mL^{-1}) was mixed with concentrated HNO_3 (32 mL) and H_2SO_4, (8 mL), the mixture was then refluxed under microwave irradiation for 9 h with an atmospheric reflux device operating at a power of 650 W. After cooling to room temperature, the mixture was placed under ultrasonication for several minutes and the pH was adjusted to 8 with Na_2CO_3 in an ice bath. The suspension was filtered through a 0.22 μm microporous membrane to remove the large tracts of GO, and a deep yellow solution was separated. The mixture was further dialyzed in a dialysis bag with a molecular weight cut off of 1 kDa, and the c-GQDs were obtained by freeze-drying.

Synthesis of h-GQDs. h-GQDs were prepared *via* a $NaBH_4$-mediated reduction reaction. Typically, $NaBH_4$ (0.27 M) was dissolved in 50 mL of UP water containing c-GQDs (0.5 mg mL^{-1}) under stirring in dark. 12 h later, above solution was purified by dialysis in a dialysis bag with a molecular weight cut off of 1 kDa and freeze-dried to result in brown h-GQDs.

Synthesis of c-GQDs-BA. BA (1 g) and c-GQDs (20 mg) were dissolved in $CHCl_3$ (10 mL). After stirring for 24 h under N_2 protection at 60 °C, the precipitate was filtered out and washed with $CHCl_3$ to remove the physically adsorbed BA molecules until the BA molecules could not be detected in the supernatant by UV-Vis-NIR spectra. The precipitate was dried in vacuum at 60°C overnight to obtain c-GQDs-BA.

Synthesis of c-GQDs-PH. PH (0.2 g) and HCl (10 μL, 38 %) were dissolved in $CHCl_3$ (10 mL), and then c-GQDs (20 mg) was added into above solution. After stirring under N_2 protection for 72 h, the precipitate was filtered out and washed with $CHCl_3$ in Soxhlet extractor for 20 h to remove the physically adsorbed PH molecules until the PH molecules could not be detected in the supernatant by UV-Vis-NIR spectra. The precipitate was dried in vacuum at 60 °C for 24 h to obtain c-GQDs-PH.

Synthesis of c-GQDs-BrPE. BrPE (0.4 g) and c-GQDs (20 mg) were dissolved in $CHCl_3$ (10 mL). After stirring under N_2 protection at room temperature in dark for 5 h, the precipitate was filtered out and washed with $CHCl_3$ to remove the physically adsorbed BrPE molecules until the BrPE molecules could not be detected in the supernatant by UV-Vis-NIR spectra. The

precipitate was dried in vacuum at 60 °C for 24 h to obtain c-GQDs-BrPE.

Synthesis of PEGylated h-GQDs (p-GQDs). h-GQDs (0.2 mg mL^{-1}) were sonicated in a solution containing mPEG-DSPE (0.2 mM) for 1 h followed by dialyzed against UP water (molecular weight cut off: 3.5 kDa), yielding a suspension of h-GQDs with non-covalent mPEG-DSPE coating, which were named as p-GQDs.[3]

Synthesis of Cy-5.5-p-GQDs. h-GQDs (0.2 mg mL^{-1}) were sonicated in a solution containing DSPE-PEG-Cy5.5 (0.2 mM) for 1 h followed by dialyzed against UP water in dark (molecular weight cut off: 3.5 kDa), yielding a suspension of h-GQDs with non-covalent DSPE-PEG-Cy5.5 coating, which were named as Cy-5.5-p-GQDs.[3]

Synthesis of PEGylated c-GQDs. c-GQDs (0.2 mg mL^{-1}) were sonicated in a solution containing mPEG-DSPE (0.2 mM) for 1 h followed by dialyzed against UP water (molecular weight cut off: 3.5 kDa), yielding a suspension of PEGylated c-GQDs with non-covalent mPEG-DSPE coating.[3]

Characterization. UV-Vis-NIR absorbance measurements were carried out on a JASCO V-550 UV-Vis-NIR spectrophotometer. TEM and HR-TEM imaging were carried out on a FEITECNAI G220 high-resolution transmission electron microscope. FT-IR measurements were carried out on a BRUKER Vertex 70 FT-IR spectrometer, 32 scans were taken with a spectral resolution of 2 cm^{-1}. ζ potential measurements were performed on Malvern Nano ZS-90 at 25℃. Fluorescence spectra were recorded on a JASCO FP-6500 spectrofluorometer. XPS measurements were performed on a Thermo Fisher Scientific ESCALAB 250Xi XPS system. For XPS analysis, sample (10 mg) was pelletized into a self-supporting wafer. O1s XPS spectra were fitted using Gaussian-Lorentzian component profiles after subtraction of a Shirley background using XPS PEAK41 software. For O1s XPS analysis, the binding energies of three peaks including C=O, COOH, and C-OH species were confined at 531.2 eV (C=O), 532.0 eV (COOH), and 533.2 eV (C-OH), respectively. The fitting was performed by fixing the peak position for individual species and the oxygen contents were determined *via* calculating the relative areas of O1s peak. Confocol imaging was carried out on a Nikon A1R confocal microscope. Fluorescence imaging was performed on an IVIS spectrum imaging system. X-ray imaging was performed on a Faxitron Multifocus preclinical X-ray imaging system.

Computational details. First-principles calculations were carried out with the Vienna ab initio simulation package.[4, 5] The interaction between ions and valence electrons was described using projector augmented wave (PAW) potentials, and the exchange-correlation between electrons was treated by using the generalized gradient approximation (GGA) in the Perdew-Burke-Ernzerhof (PBE) form.[6] To achieve the accurate density of electronic states, the plane wave cutoff energy was set to 480 eV, Γ-point was used for calculated molecules. Ionic relaxations were carried out under the conventional energy (10^{-4} eV) and force (0.01 eV/Å) convergence criteria. In the present study, a graphene structure contains 30 carbon atoms was applied for all the calculations, where the unsaturated fringe carbon atoms were saturated with H atoms. The bond dissociation

energy of O-H group in different calculated models was obtained according to the following expression:

$\Delta E = E(O) + E(H) - E(OH)$, where ΔE was the bond dissociation energy, E(O) represented the substrate energy after dissociating the H atom, E(H) was the calculated energy of H atoms, and E(OH) was the substrate energy before dissociating the H atoms. All the energy of E(O), E(H), and E(OH) was calculated with VASP without any correction.

DPPH· scavenging activity of various GQDs. Freshly prepared stock DPPH· methanolic solution was mixed with the aqueous solutions of various GQDs (60 μL) and incubated in dark. The experimental concentrations of DPPH· in above systems were 100 μM. 2 h or 24 h later, UV-Vis-NIR absorption spectra of above solutions of DPPH· were monitored, and the absorbance at 515 nm was recorded. Finally, DPPH· scavenging efficacy was calculated by using the ratio of neutralized DPPH· to the overall DPPH· radicals.

$ABTS^{+}$· scavenging activity of various GQDs. Firstly, ABTS (7 mM) was dissolved in water containing $K_2S_2O_8$ (2.45 mM) to produce $ABTS^{+}$·, and above mixture was kept in dark at room temperature for 24 h before use, which was named as stock solution. Secondly, the concentration of $ABTS^{+}$· of above stock solution was adjusted to 34.5 μM with PBS (10 mM, pH 7.4). After the addition of various GQDs, above solutions were further incubated in dark for another 30 min. Thirdly, UV-Vis-NIR absorption spectra of above diluted solutions of $ABTS^{+}$· were monitored, and the absorbance at 734 nm was recorded. $ABTS^{+}$· scavenging efficacy was calculated by using the ratio of neutralized $ABTS^{+}$· to the overall $ABTS^{+}$·.

Superoxide radicals scavenging activity of various GQDs. Superoxide radicals scavenging activity was explored by using SOD assay kit (Sigma-Aldrich). Experiments were carried out following the protocol provided by the manufacturers. Typically, each sample (20 μL) containing various GQDs was mixed with the WST-1 working solution (160 μL). Then, xanthine oxidase solution (20 μL) was added to each well. 20 min later, the absorbance at 450 nm was measured using a Bio-Rad model-680 microplate reader.

Hydroxyl radicals scavenging activity of various GQD. Hydroxyl radicals scavenging activity was investigated by using a hydroxyl radical antioxidative capacity assay kit (Cell Biolabs). Experiments were performed following the protocol provided by the manufacturers. Typically, each sample (20 μL) containing various GQDs was mixed with fluorescent working solution (140 μL). After hydroxyl radical initiator (20 μL) was added to above samples, Fenton reagent (20 μL) was added immediately. After shaking for 15 s and incubating for 30 min in dark, fluorescence was measured using a Bio-Rad model-680 microplate reader.

Cell culture. Human embryonic kidney 293T (HEK293T) cells were purchased from the American Type Culture Collection (ATCC) and cultured at 37 °C under 5% CO_2. Media was RPMI-1640 containing FBS (10%), penicillin (100 U mL^{-1}), and streptomycin (100 mg mL^{-1}). Cells were digested by trypsin and re-suspended in fresh media before plating.

MTT assay. HEK-293T cells were seeded in a 96-well plate with a density of 10^5 per well. 12 h later, p-GQDs with different

concentrations were added into above medium. 24 h later, cells were treated with MTT for another 4 h. DMSO was added to dissolve the formazan crystals, and Bio-Rad model-680 microplate reader was used to measure the absorbance at 490 nm with 570 nm as a reference.

Cellular uptake. HEK-293T cells with a density of 10^5 were seeded in a 6-well plate. After 24 h attachment, cells were treated with p-GQDs with different concentrations. 2 h later, cells were washed with saline (0.9% NaCl solution), and cellular fluorescence imaging was performed on a Nikon A1R confocal microscope.

***In vitro* ROS scavenging ability of p-GQDs.** HEK-293T cells were seeded in a 6-well plate with a density of 10^5 per well. 24 h later, cells were incubated with p-GQDs for 4 h. To remove excess p-GQDs, cells were washed several times with saline. Afterwards, LPS (1 μg mL^{-1}) was added and incubated for another 6 h to stimulate the generation of intracellular ROS. Finally, cells were incubated with DCFH-DA (10 μM), and cellular fluorescence intensity an related imaging was monitored *via* flow cytometry analysis and confocal imaging, respectively.

Murine model of rhabdomyolysis-induced acute kidney injury. All animal experiments were performed according to the NIH guidelines for the care and use of laboratory animals (NIH Publication No. 85-23 Rev. 1985) and approved by the Jilin University Animal Care and Use Committee. Balb/c mice weighted about 25 g were obtained from Laboratory Animal Centre of Jilin University (Changchun, China). Before the initiation of AKI model, all mice were deprived of water but had access to food for 16 h. At the end of the water restriction, glycerol in saline (50%) was intramuscularly injected into each hind limb of the mice with a dosage of 8 mL kg^{-1}. This time point was defined as the initiation of AKI. Afterwards, mice were given free access to water and food. For fluorescence imaging studies, Balb/c nude mice weighted about 18 g were used in all the experiments.

***In vivo* and *ex vivo* fluorescence imaging of Cy-5.5-p-GQDs.** For fluorescence imaging studies, AKI nude mice were intravenously injected with Cy-5.5-p-GQDs (0.8 mg kg^{-1} Cy5.5). Afterwards, mice were anesthetized and imaged at different expected time points. Moreover, mice were sacrificed and major organs were collected and imaged. Images were analyzed *via* ImageJ Software.

Bio-distribution analysis. For bio-distribution analysis, AKI nude mice were intravenously injected with Cy-5.5-p-GQDs (0.8 mg kg^{-1} Cy5.5). Fluorescence from Cy5.5-p-GQDs allowed an *ex vivo* quantitative analysis *via* tissue homogenates.[7, 8] After mice were sacrificed, main organs including heart, liver, spleen, lung, and kidney were harvested and weighted. Then, above organs were homogenized *via* a tissue homogenizer and completely digested by a lysis buffer. After centrifugation, fluorescent signals of above supernatant from organ homogenate were determined and normalized using the control mouse organ with an excitation of 670 nm. For the calibration curve, Cy5.5-p-GQDs with different amounts were added into the digested supernatant of organ homogenate from control mice, and relative fluorescence intensities were determined. Finally,

the amounts of Cy5.5-p-GQDs distributed in each tissue were calculated according to the standard curve.

Imaging of Cy5.5-p-GQDs in kidneys. AKI mice were intravenously injected with Cy-5.5-p-GQDs (0.8 mg kg^{-1} Cy5.5) and kidneys were harvested at various expected time points and stored at -20°C for cryostat sectioning. Frozen kidney slices of 5 μm thickness were washed with cold saline. Then, a cover glass was applied to each slide, and confocal imaging was performed on a Nikon A1R confocal microscope.

Antioxidative therapy of AKI mice. 2 h after the AKI model induction, different treatments were carried out on healthy mice and AKI mice, respectively. In detail, group 1 was healthy mice treated with saline, group 2 was healthy mice treated with p-GQDs (10 mg kg^{-1}), group 3 was AKI mice treated with saline, and group 4 was AKI mice treated with p-GQDs (10 mg kg^{-1}). 1 d after various treatments, mouse renal functions in different groups were explored.

Non-invasive X-ray imaging of urinary system for kidney function evaluation. Mice in above 4 groups were anesthetized at first. A catheter was inserted into the tail vein before the mouse was transferred into the imaging chamber and placed in its supine position. X-ray imaging was carried out on a Faxitron Multifocus preclinical X-ray imaging system with the following parameters: voltage: 45 kV, exposure time: 10 s, and field of view: 100 mm×150 mm. X-ray images were taken before and after intravenous injection of Iopromide (0.5 mL, Ultravist™, Bayer) with an iodine concentration of 370 mg mL^{-1}. Relative X-ray signal intensities were analyzed *via* Sante DICOM Editor 4 software.

Histological analysis. Harvested tissues including heart, liver, spleen, lung, and kidney were fixed with paraformaldehyde (4%), dehydeated, embedded in paraffin, sectioned, and stained with hematoxylin and eosin (H&E). Slides were observed on an Olympus BX-51 optical system.

Body weight measurement and survival rate study. Survival curve and body weight of AKI mice were monitored for 7 days after different treatments. According to the guidelines on animal welfare, body weight loss in a mouse higher than 15% was considered to be death for the purpose of drawing the survival curve.

Analysis of renal tissues after treatment. Kidneys from above groups were frozen and stored at -80 °C. Kidney homogenates were prepared in accordance with the protocols of different assays. The degrees of lipid peroxidation were determined with a malondialdehyde (MDA) assay kit (Abcam).

Hematology analysis and blood biochemical assay. Blood of mice in all groups were collected at expected time point, and the samples were used to perform hematology analysis and blood biochemical assay. Blood from the mice without any treatments were defined as the control group. Blood from the mice different periods after intravenous injection of p-GQDs (10 mg kg^{-1}) were defined as the test groups.

Statistical analysis. All data were expressed as mean ± standard deviation (SD) and performed in at least 3 specimens. A P value <0.05 was considered statistically significant. Statistical analysis was performed by two-tailed *t*-test.

Ⅱ. Figures

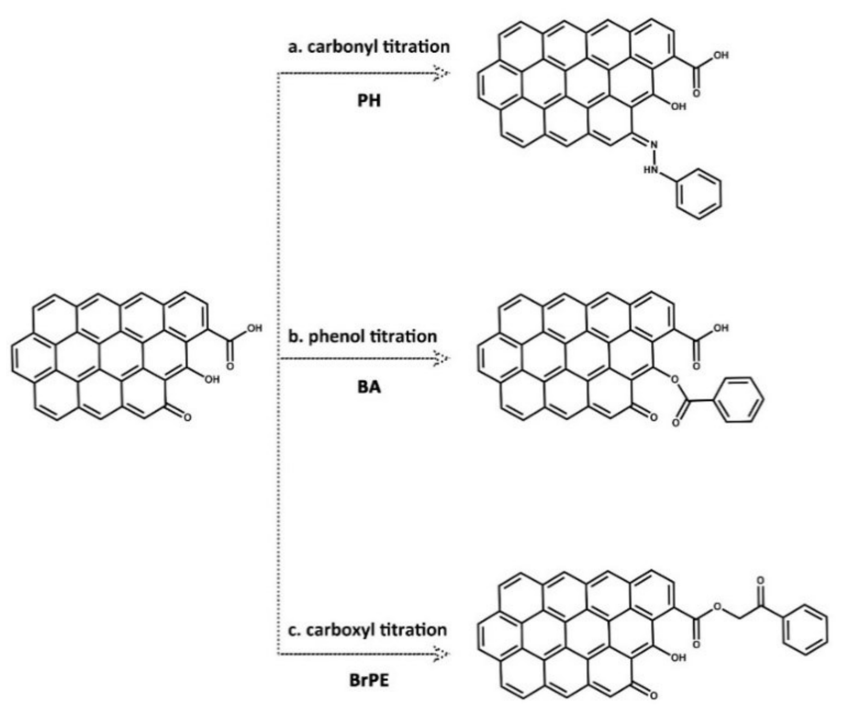

Fig. S1. Schematic illustration of the chemical titration processes for C=O (a), C−OH (b), and COOH (c) on c-GQDs.

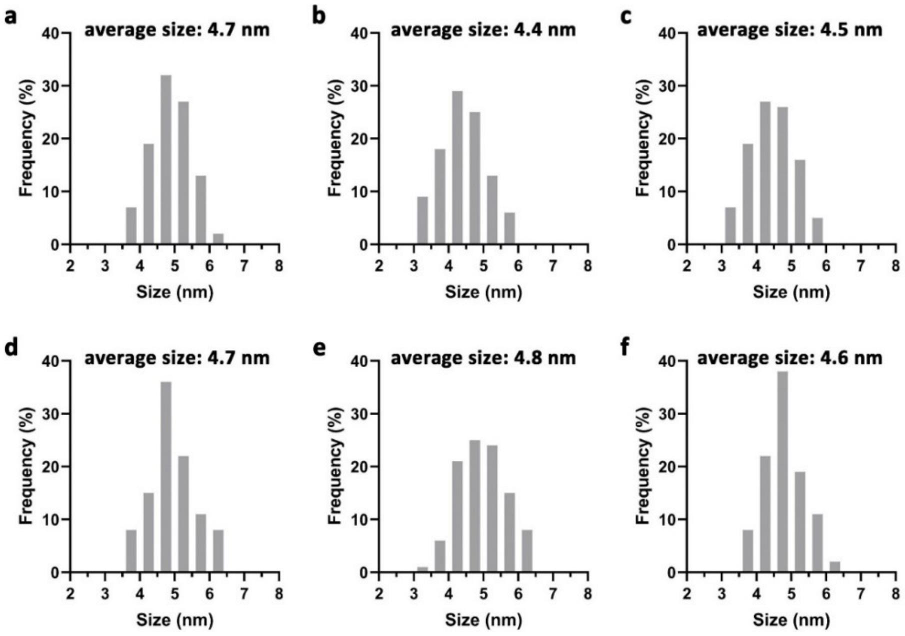

Fig. S2. Size distributions of c-GQDs (a), h-GQDs (b), p-GQDs (c), c-GQDs-PH (d), c-GQDs-BA (e), and c-GQDs-BrPE (f).

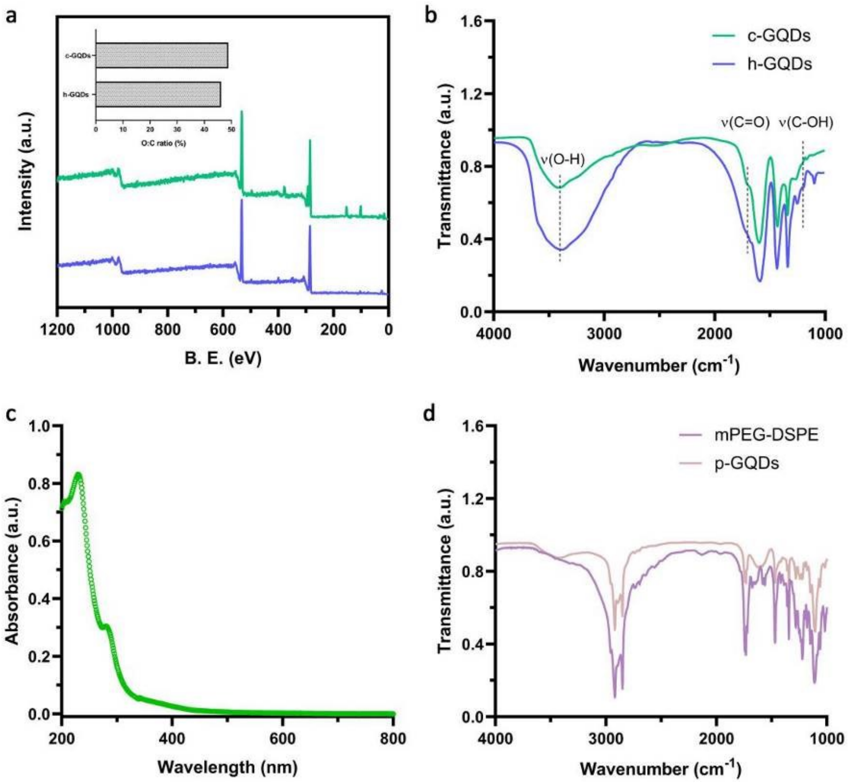

Fig. S3. XPS spectra of c-GQDs and h-GQDs (a). Inset: the O/C atomic ratios of c-GQDs and h-GQDs. FT-IR spectra of c-GQDs and h-GQDs (b). UV-Vis-NIR spectrum of p-GQDs (c). FI-IR spectra of m-PEG-DSPE and p-GQDs (d).

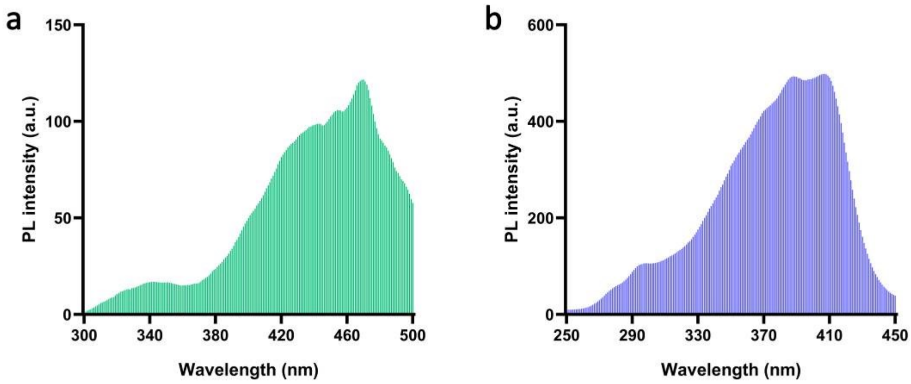

Fig. S4. PLE spectra of c-GQDs (a) and h-GQDs (b). Emissions: 515 nm (a) and 465 nm (b).

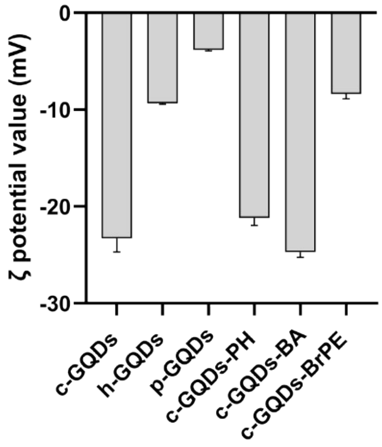

Fig. S5. ζ potential values of various GQDs in water. Error bars represent standard deviation from the mean (n=3).

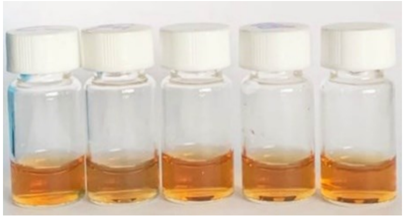

Fig. S6. Photograph of various GQDs dissolved in water (2 mg mL^{-1}). From left to right: c-GQDs, h-GQDs, c-GQDs-PH, c-GQDs-BA, and c-GQDs-BrPE.

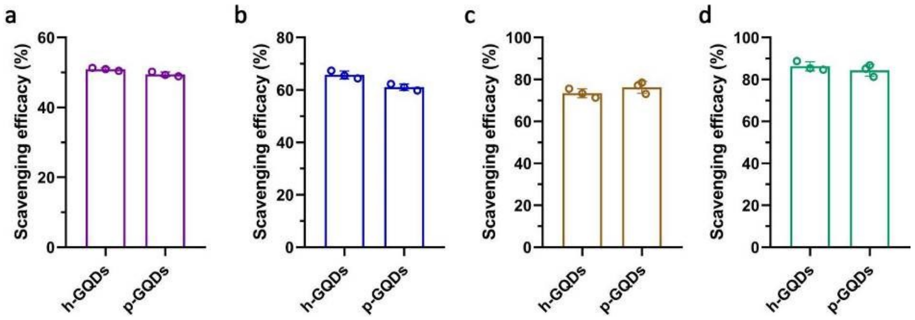

Fig. S7. The scavenging efficacies of DPPH· (a), $ABTS^{+}$· (b), superoxide radicals (c), and hydroxyl radicals (d) of h-GQDs, and p-GQDs, respectively. Error bars represent standard deviation from the mean (n=3).

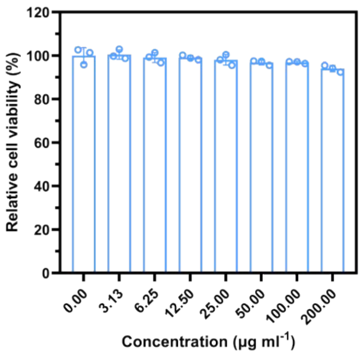

Fig. S8. Viabilities of HEK-293T cells treated with p-GQDs (24 h). Error bars represent standard deviation from the mean (n=3).

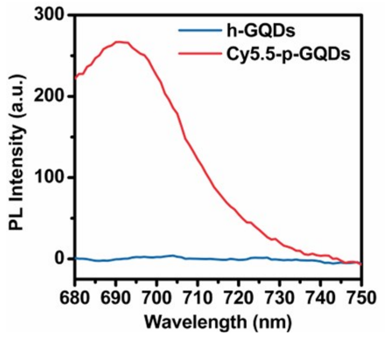

Fig. S9. PL spectra of h-GQDs and Cy5.5-p-GQDs. The excitation wavelength was 670 nm.

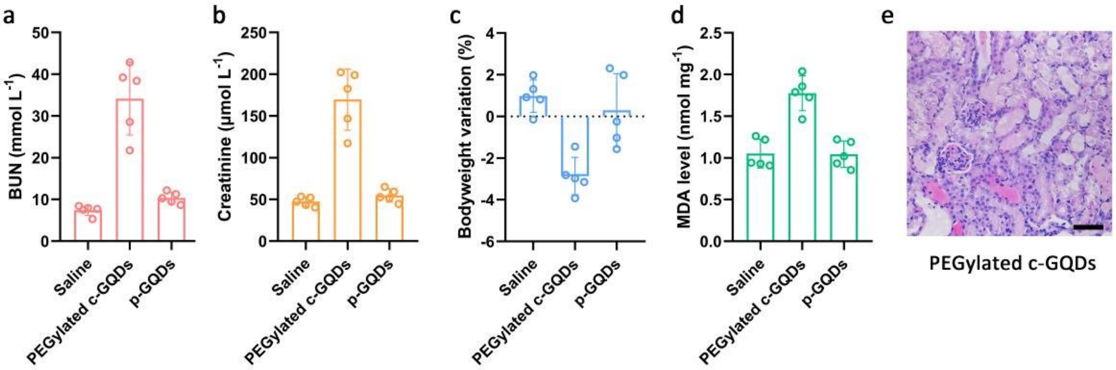

Fig. S10. BUN levels (a) and CRE levels (b) in the blood from saline-treated healthy mice, p-GQDs-treated AKI mice, and PEGylated c-GQDs-treated AKI mice one day post-initiation of AKI. Bodyweight changes of mice after various treatments one day post-initiation of AKI (c). MDA levels in the homogenates of kidneys from saline-treated healthy mice, p-GQDs-treated AKI mice, and PEGylated c-GQDs-treated AKI mice at one day post-initiation of AKI (d). Error bars represent standard deviation from the mean (n=5). H&E staining images of kidney tissue collected from PEGylated c-GQDs-treated AKI mice one day post-initiation of AKI (e). Scale bars are equal to 50 μm.

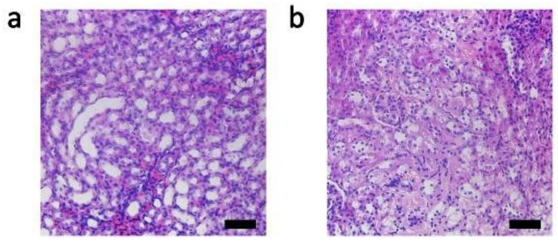

Fig. S11. H&E staining images of kidney tissues collected from p-GQDs-treated AKI mice at 3 (a) and 7 (b) days post-initiation of AKI. Scale bars are equal to 50 μm.

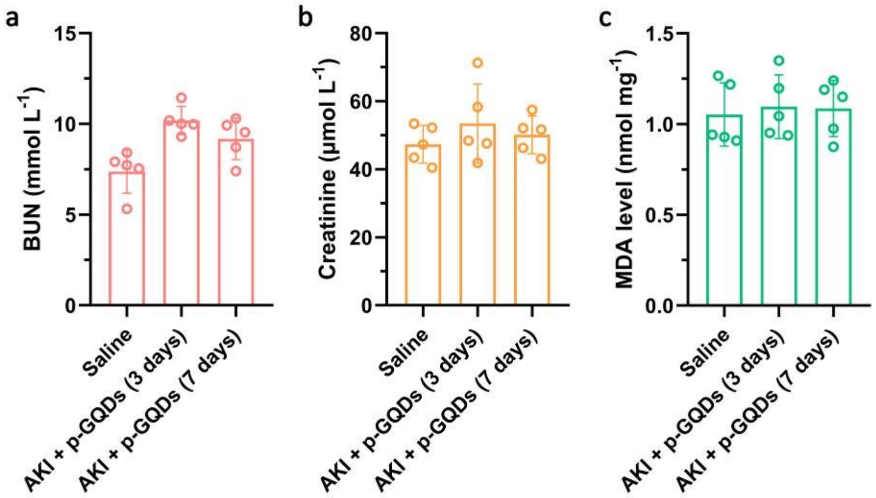

Fig. S12. BUN levels (a) and creatinine levels (b) in the blood from saline-treated healthy mice and p-GQDs-treated AKI mice at 3 and 7 days post-initiation of AKI. MDA levels in the homogenates of kidneys from saline-treated healthy mice and p-GQDs-treated AKI mice at 3 and 7 days post-initiation of AKI (c). Error bars represent standard deviation from the mean (n=5).

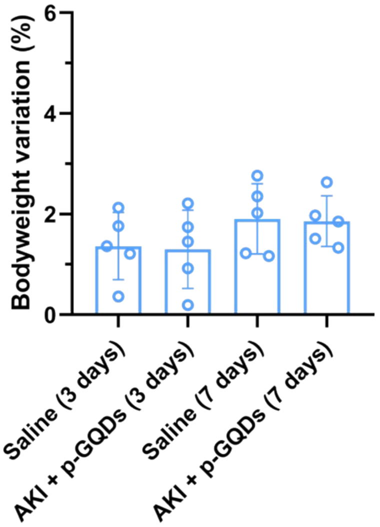

Fig. S13. Bodyweight changes of saline-treated healthy mice and p-GQDs-treated AKI mice at 3 and 7 days post-initiation of AKI. Error bars represent standard deviation from the mean (n=5).

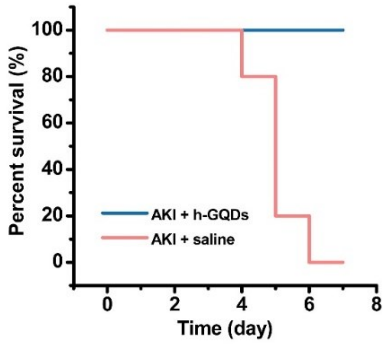

Fig. S14. Survival curves of AKI mice after various treatments. Error bars represent standard deviation from the mean (n=5).

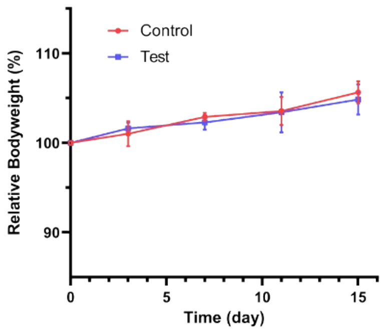

Fig. S15. Time-dependent bodyweight changes of the healthy mice after intravenous injection of p-GQDs. Error bars represent standard deviation from the mean (n=3).

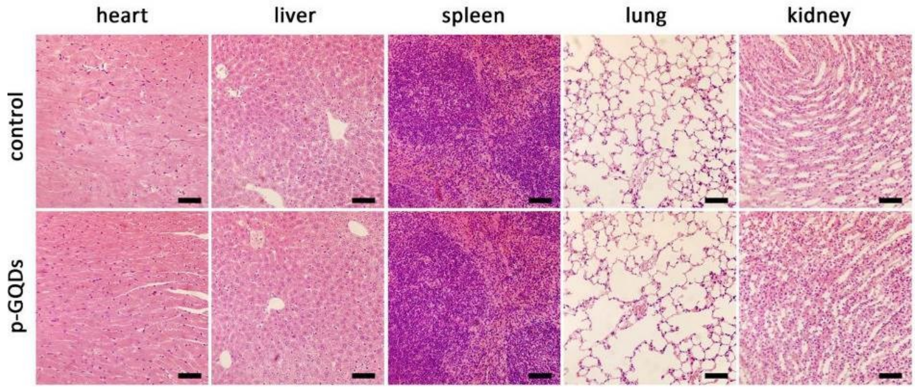

Fig. S16. H&E-stained images of major organs including heart, liver, spleen, lung, and kidneys harvested from healthy mice 15 days after intravenous injection of p-GQDs. Scale bars are equal to 50 μm.

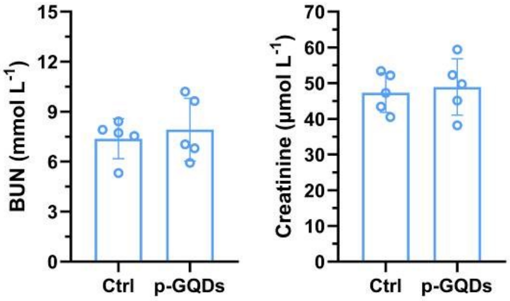

Fig. S17. Serum levels of blood BUN and CRE of healthy mice after intravenous injection of p-GQDs. Blood was collected at 15 days after injection. Error bars represent standard deviation from the mean (n=5).

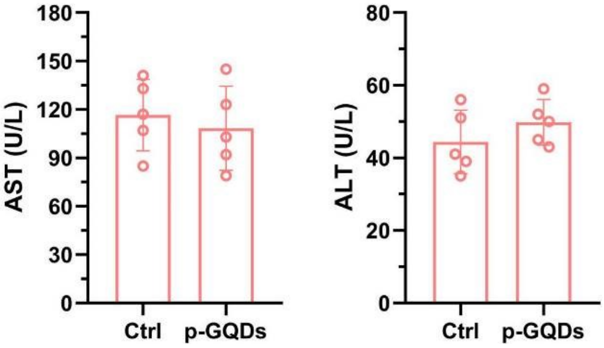

Fig. S18. Serum levels of AST, and ALT of healthy mice after intravenous injection of p-GQDs. Blood was collected at 15 days after injection. Error bars represent standard deviation from the mean (n=5).

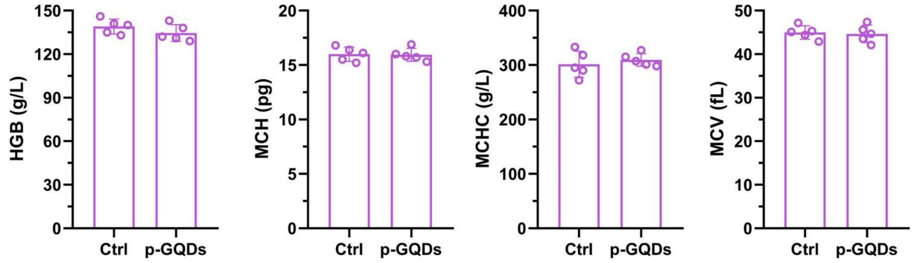

Fig. S19. Serum levels of hemoglobin (HGB), mean corpuscular hemoglobin (MCH), mean corpuscular hemoglobin concentration (MCHC), and mean corpuscular volume (MCV) of healthy mice after intravenous injection of p-GQDs. Blood was collected at 15 days after injection. Error bars represent standard deviation from the mean (n=5).

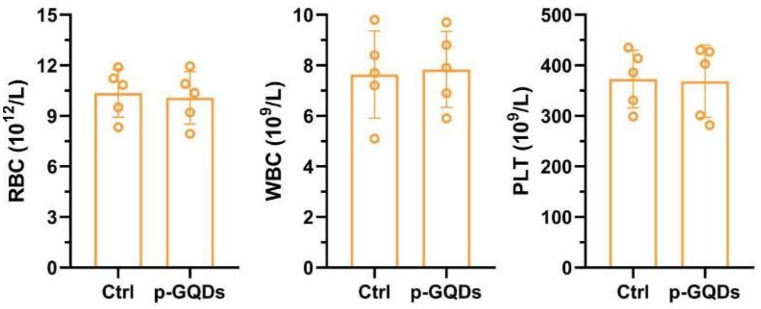

Fig. S20. Serum levels of red blood cells (RBC), white blood cells (WBC), and platelets (PLT) of healthy mice after intravenous injection of p-GQDs. Blood was collected at 15 days after injection. Error bars represent standard deviation from the mean (n=5).

Ⅲ. References

1. Sun, H.; Zhao, A.; Gao, N.; Li, K.; Ren, J.; Qu, X., Deciphering a Nanocarbon-Based Artificial Peroxidase: Chemical Identification of the Catalytically Active and Substrate-Binding Sites on Graphene Quantum Dots. *Angew. Chem. Int. Ed.* **2015,** *54* (24), 7176-7180.
2. Zhou, Y.; Sun, H.; Wang, F.; Ren, J.; Qu, X., How functional groups influence the ROS generation and cytotoxicity of graphene quantum dots. *Chem. Commun.* **2017,** *53* (76), 10588-10591.
3. Liu, Z.; Davis, C.; Cai, W.; He, L.; Chen, X.; Dai, H., Circulation and long-term fate of functionalized, biocompatible single-walled carbon nanotubes in mice probed by Raman spectroscopy. *Proc. Natl. Acad. Sci. U. S. A.* **2008,** *105* (5), 1410.
4. Kresse, G.; Furthmüller, J., Efficiency of ab-initio total energy calculations for metals and semiconductors using a plane-wave basis set. *Comp. Mater. Sci.* **1996,** *6* (1), 15-50.
5. Surendranath, Y.; Kanan, M. W.; Nocera, D. G., Mechanistic Studies of the Oxygen Evolution Reaction by a Cobalt-Phosphate Catalyst at Neutral pH. *J. Am. Chem. Soc.* **2010,** *132* (46), 16501-16509.
6. Perdew, J. P.; Burke, K.; Ernzerhof, M., Generalized Gradient Approximation Made Simple. *Phys. Rev. Lett.* **1996,** *77* (18), 3865-3868.
7. Wan, H.; Yue, J.; Zhu, S.; Uno, T.; Zhang, X.; Yang, Q.; Yu, K.; Hong, G.; Wang, J.; Li, L.; Ma, Z.; Gao, H.; Zhong, Y.; Su, J.; Antaris, A. L.; Xia, Y.; Luo, J.; Liang, Y.; Dai, H., A bright organic NIR-II nanofluorophore for three-dimensional imaging into biological tissues. *Nat. Commun.* **2018,** *9* (1), 1171.
8. Gao, R.; Mei, X.; Yan, D.; Liang, R.; Wei, M., Nano-photosensitizer based on layered double hydroxide and isophthalic acid for singlet oxygenation and photodynamic therapy. *Nat. Commun.* **2018,** *9* (1), 2798.
